# A Novel Hard Decision Based Simultaneous Target Tracking and Classification Approach

**DOI:** 10.3390/s18020622

**Published:** 2018-02-19

**Authors:** Wen Cao, Meng Hui, Qisheng Wu

**Affiliations:** School of Electronics and Control Engineering, Chang’an University, Xi’an 710064, China; ximeng@chd.edu.cn (M.H.); qshwu@chd.edu.cn (Q.W.)

**Keywords:** simultaneous tracking and classification, joint target tracking and classification, hard decision, sequential probability ratio test

## Abstract

Methods dealing with the problem of Joint Tracking and Classification (JTC) are abundant, among which Simultaneous Tracking and Classification (STC) provides a modularized scheme solving tracking and classification subproblems simultaneously. However, there is no explicit hard decision on the class label but only soft decision (class probability) is provided. This does not fit many practical cases, in which a hard decision is urgently needed. To solve this problem, this paper proposes a Hard decision-based STC (HSTC) method. HSTC takes all the decision error rate, timeliness, and estimation error into account. Specifically, for decision, the sequential probability ratio test is adopted due to its nice properties and also the adaptability to our situation. For estimation, by utilizing the two-way information exchange between the tracker and the classifier, we propose flexible three tracking schemes related to decision. The HSTC tracking result is divided into three parts according to the time of making the hard decision. In general, the proposed HSTC method takes advantage of both SPRT and STC. Finally, two illustrative JTC examples with hard decision verify the effectiveness of the the proposed HSTC method. They show that HSTC can meet the demand of the problem, and also has the performance superiority in both decision and estimation.

## 1. Introduction

Target tracking and classification are critical in battlefield surveillance systems [1,2,3,4,5,6,7,8]. Traditionally, they are treated separately using their respective data and techniques: tracking is usually based on kinematic data while classification relies on attribute data. Meanwhile, since they involve continuous and discrete valued uncertainties respectively, their solutions are different. Recently, solving these two problems jointly has attracted much attention. This is preferable since in many practical problems involving both tracking and classification, the two problems are interrelated: tracking may affect classification by providing flight envelope information for different classes while classification may affect tracking via selecting appropriate class-dependent kinematic models. It is easy to realize that for such problems, tracking and classification should be handled jointly, and the performance of both could be improved by effectively utilizing their mutual-effect.

For solving the Joint Tracking and Classification (JTC) problem, many existing methods ignore the interdependence between tracking and classification [9,10] or only considering the one-way dependence [11,12]. Specifically, in the former, tracking and classification proceed separately. The tracker utilizes the kinematic information for state estimation and the classifier uses identity information for classification, respectively. In the latter, tracking and classification are handled within two-stages considering their mutual-effect to some extent. This can be further classified into two types: tracking then classification (T-then-C) [11,13,14,15,16] and classification then tracking (C-then-T) [12,17]. In T-then-C, it is considered that identity relies heavily on accurate state estimation. The basic idea is that each target class has its own motion model which can be seen as priors. Thus after tracking, the estimated kinematic state is compared with the flight envelops of known classes for class identification. While in C-then-T, it is considered that target type knowledge could help improve tracking performance by, e.g., assisting correct data association and selecting appropriate target models.

Some other researches realize the importance of two-way dependence and one prevailing strategy is the density based method in Bayesian framework, which aims to obtain the posterior joint density-probability of target state and class [18,19,20]. However, this results in heavy computational complexity due to its essence of density inference. Actually, JTC is a point estimation-based rather than density-based problem, and density estimation is even harder than the joint problem itself. We should avoid intermediate subproblems that are even harder than the original problem [21]. For the JTC problem, a novel Joint Decision and Estimation (JDE) framework proposed by Li [22] has provided a good joint solution for problems involving interdependent decision and estimation. It has the potential of arriving at the globally optimal solution. The power of this framework has been demonstrated in [21,23,24,25]. JDE framework belongs to another new and superior framework, which is not considered in this paper. For multiple targets, [26,27] propose a variational approach to simultaneous tracking and classification of multiple objects, whose superiority has been effectively verified. In this paper, however, we consider the problem of single target joint tracking and classification without measurement uncertainties.

In [28], a Simultaneous Tracking and Classification (STC) method was proposed within a modularized scheme. It derives the tracker and classifier simultaneously by utilizing the marginal pdf-pmf of the target state and class directly. A multiple-model filter and a Bayesian classifier are presented together using multisensor data. Under the linear Gaussian assumption, the STC tracking and classification results are obtained based on point inference rather than density inference.

In many existing literatures, there is no explicit hard decision on the class label but only soft decision (class probability) is provided. However, in many real applications, an explicit hard decision is urgently needed because decision usually leads to action. Take air warning system as an example. Suppose that there are two targets in the field of interest, a fighter and an airliner. Our goal is to recognize and track the two targets simultaneously using data from multiple sensors. Giving an alarm signal if a fighter is recognized. In this case, a hard decision is urgently needed. A wrong decision may either lead to false alarm or miss, both of which will bring loss. Another common example is a ground target tracking and identification system. Suppose that two targets, a tank and a school-bus are moving in the crossroads with different motion models, we want to destroy the tank and let the school-bus pass by. Hard decision is necessary and urgent since we want to destroy the tank as soon as possible.

In view of the above, a hard decision is critical in a joint tracking and classification problem. For such a decision, both the timeliness of decision-making and the corresponding decision error should be taken into account. Sequential testing is natural for classification where observations are obtained sequentially. A sequential test consists of a stopping rule and a final decision, whose performance is usually measured by the average sample number (ASN) given allowable decision error probabilities. In view of the above, we propose to use the well known sequential probability ratio test (SPRT) [29] due to its nice properties: SPRT is optimal in the sense that it minimizes the ASN under both hypotheses simultaneously among all tests of the same allowable error probabilities [30]. Besides, SPRT is also adaptable to our JTC problem with hard decision. In many JTC problems, the true target class remains constant. This consists with the basic assumption of SPRT, in which the true hypothesis is assumed to be unchanged over time.

For tracking, considering that tracking and classification are interactive in JTC, better tracking schemes require handling the tracking problem by utilizing their two-way effect. Thus, this paper proposes flexible decision-related tracking strategies. According to the time instant of the hard decision, i.e., before hard decision, at hard decision, and after hard decision, we provide three different tracking results. Finally, all the decision error, decision timeliness, and the estimation performance are considered. In general, the proposed tracking schemes not only meet the demand of the JTC problem but also has nice properties. It takes advantage of the STC method in handling the mutual-effect between decision and estimation within the point inference framework, furthermore, it accounts for the relationship between the hard decision and estimation.

The main contributions of this paper are as follows: (a) We clearly formulate the practical JTC problem in which both the estimation of the target state and the hard decision on the target class are required. (b) A Hard decision-based STC (HSTC) method is creatively proposed with SPRT being the decision criterion. HSTC not only satisfies the practical requirements but also superior in performance. It takes advantage of both SPRT and STC, and adapts to our problem. It considers the decision error, decision timeliness, estimation accuracy, and also utilizes the mutual information exchange between tracking and classification. (c) Two illustrative examples demonstrate the superiority of the proposed HSTC method.

This paper is organized as follows. Section 2 formulates the JTC problem with hard decision. Section 3 reviews the existing STC method. Section 4 as the main part of the paper proposes the HSTC method. Both the HSTC classification and tracking results are presented. In Section 5, two numerical examples are used to illustrate the effectiveness of the HSTC method. Conclusions are made in Section 6.

## 2. Problem Formulation

In this paper, we consider a simple but representative joint tracking and classification problem. Suppose there is only one target in the field of interest, and it has two possible classes: a fighter and an airliner. We want to track the target and identify its class using all available data. Assume that different target classes have different maneuverabilities: the fighter has larger maneuverability than the airliner, and there is no measurement origin uncertainty. Then, denote by $x_{k}$ the target state and $c_{i}$ the target class, our goal is to obtain $\left\{x_{k},c_{i}\right\}$ jointly using $z^{k}$, where $z^{k}$ denotes the measurements up to time *k*.

Assume that a target of class *i* has $r_{i}$ possible models and the motion model corresponding to class *i* can be written as

(1) $$p\left(x_{k}|x_{k-1},m_{k}^{ij}\right),\;i=1,\cdots ,N_{c}$$

where the total number of target class is $N_{c}$, $x_{k}$ denotes the target state at time *k*, and $m_{k}^{ij}$ is the *j*th motion model of a class *i* target at time *k*.

Denote the model set for class *i* as

$$M^{i}=\left\{m^{i1},m^{i2},\cdots ,m^{ir_{i}}\right\}$$

The transition probability matrix (TPM) for class *i* is

$$\Pi^{i}={\left[p_{lj}^{i}\right]}_{l,j=1}^{r_{i}}=\left[p\left(m_{k}^{ij}|m_{k-1}^{il}\right)\right]$$

and the initial model probability for class *i* is

$$\mu_{0}^{i}={\left[\mu_{0}^{i1},\mu_{0}^{i2},\cdots ,\mu_{0}^{ir_{i}}\right]}^{\prime }.$$

Assuming that the target state evolves according to the following linear dynamics:

(2) $$x_{k+1}=F_{k}^{ij}x_{k}+G_{k}^{ij}u_{k}^{ij}+\Gamma_{k}^{ij}w_{k}^{ij}$$

where $u_{k}^{ij}$ is the deterministic input at time *k*, $w_{k}^{ij}$ is assumed to be zero-mean white Gaussian process noise with known variance $Q_{k}^{ij}$, $F_{k}^{ij}$ is the state transition matrix, $G_{k}^{ij}$ and $\Gamma_{k}^{ij}$ are the gain matrices of input and process noise, respectively, and the superscript ij denotes a quantity for the *j*th model of a class *i* target.

The kinematic measurement is denoted by $z_{k}$. Under the linear measurement assumption, we have

(3) $$z_{k}=H_{k}x_{k}+v_{k}$$

where $H_{k}$ is the measurement matrix, and $v_{k}$ is assumed to be zero-mean white Gaussian noise with known variance $R_{k}$.

## 3. Existing Simultaneous Tracking and Classification Method

To obtain the target state $x_{k}$ and class $c_{i}$ jointly, the most fundamental solution is to calculate the joint density-probability of the target state and class [28]:

(4) $$p(x_{k},c_{i}|z^{k})$$

Most existing methods calculate $p(x_{k},c_{i}|z^{k})$ first and then marginalize it to obtain the target state and class:

$$\begin{array}{cc} p(x_{k}|z^{k}) & =\sum_{i}p\left(x_{k},c_{i}|z^{k}\right)\\ p\{c_{i}|z^{k}\} & =\int p\left(x_{k},c_{i}|z^{k}\right)dx_{k} \end{array}$$

To obtain the joint density $p(x_{k},c_{i}|z^{k})$, many numerical calculation methods are adopted. Different from these, in the STC method [28], the simultaneous pdf-pmf:

(5) $$\left\{\begin{array}{c} p(x_{k}|z^{k})\\ p\{c_{i}|z^{k}\} \end{array}\right.$$

is used directly rather than calculate $p(x_{k},c_{i}|z^{k})$ first and then marginalize to get $p(x_{k}|z^{k})$ and $p(c_{i}|z^{k})$. With this method, both the tracker and the classifier can be obtained simultaneously by accounting for the mutual information exchange between them [28]. Note that under the linear Gaussian assumption, STC requires only the first two moments of the joint distribution of the target state and class. This avoids the complex density calculation, and makes it computation efficient.

## 4. Hard Decision Based Simultaneous Tracking and Classification

As is explained in Introduction, how can we solve such a joint tracking and classification problem involving hard decision? For decision, a hard and timeliness decision is needed, which explicitly determine the target class. For estimation, the effect that the hard decision may exert should be taken into account. In the following, they will be presented in detailed.

### 4.1. HSTC Decision

To begin with, sequential testing procedures are preferable since measurements come sequentially. A sequential test contains a stopping rule and a final decision to achieve a trade-off between sampling size and decision accuracy. For JTC problem with hard decision case, the class label should be identified as quickly as possible and the decision error should be as small as possible.

As is well known that SPRT has the nice property [29] that when the type I and type II errors are controlled, the expected sample size under both hypotheses are simultaneously minimized among all the tests. This matches the requirements for our problem exactly. Besides, as mentioned in the Introduction part, the target type is always the same with no class switching problem over time. SPRT fits this situation perfectly. Thus it is adopted in the decision part of our HSTC method.

#### Overview of SPRT

In this paper, we assume that hypothesis and target class are one-to-one correspondence:

$$“H^{i}”:\;\mathrm{target}\;\mathrm{class}\;“c_{i}”\;\mathrm{is}\;\mathrm{decided}$$

Following SPRT, once a piece of new data arrives, the class-likelihood will be output by the filters, given by:

(6) $$P\left\{c_{i}|z^{k}\right\}=\frac{1}{\delta }p\left(z_{k}|z^{k-1},c_{i}\right)P\left\{c_{i}|z^{k-1}\right\}$$

in which $p(z_{k}|z^{k-1},c_{i})$ is the measurement likelihood at time *k*, which will be presented in detail later. δ is a normalization factor, and $P\{c_{i}|z^{k-1}\}$ is the class probability based on $z^{k-1}$.

Before the hard decision is made, only the probability of target class is available. Therefore, the HSTC classifier is given by (6). Following SPRT, to make a hard decision, we need to calculate the log-likelihood ratio (LLR), given by

(7) $$\begin{array}{c} L\left(z^{k}\right)=\log\frac{P\{c_{1}|z^{k}\}}{P\{c_{2}|z^{k}\}} \end{array}$$

(8) $$\begin{array}{c} =\log\frac{p(z_{k}|z^{k-1},c_{1})}{p(z_{k}|z^{k-1},c_{2})}+\log\frac{P\{c_{1}|z^{k-1}\}}{P\{c_{2}|z^{k-1}\}} \end{array}$$

Assume that there is no prior information about target class, we have $p\left\{c_{1}|z^{0}\right\}=p\left\{c_{2}|z^{0}\right\}=0.5$, and thus the $L(z^{k})=0$ initially. Based on this, the class-likelihood ratio is equivalent to the class posterior probability ratio when it is updated recursively. That is why we use the posterior class probability here, which is available at every step from the soft decision.

Based on the above, the decision rule in HSTC is given by

(9) $$\begin{array}{cc} L\left(z^{k}\right)\geq \tau_{1}\; & \mathrm{declare}\;c_{1}\\ \tau_{2}<L\left(z^{k}\right)<\tau_{1}\; & \text{soft decision}\\ L\left(z^{k}\right)\leq \tau_{2}\; & \mathrm{declare}\;c_{2} \end{array}$$

where

$$\begin{array}{cc} \tau_{1} & =\log\frac{1-\beta }{\alpha },\;\tau_{2}=\log\frac{\beta }{1-\alpha },\\ \;\alpha & =P\left\{“c_{1}”|c_{2}\right\},\;\beta =P\left\{“c_{2}”|c_{1}\right\} \end{array}$$

**Remark 1:** *In this paper, we consider the target classification with two possible classes, as is presented in the problem formulation part. Therefore, we adopt the SPRT for hard decision due to its nice properties in handling the binary hypothesis testing. Actually, the proposed HSTC method can extended to multi-classes by replacing the SPRT method with Armitage test [31]. Armitage ’s test is a generalization of Wald’s SPRT to the multi-hypothesis case. It can control the whole matrix of decision error probabilities and has widespread applications due to its simplicity.*

### 4.2. HSTC Estimation

Since estimation is closely related to decision in the JTC problem, we should fully consider their mutual influence. In the following, we first analyze the HSTC estimation regardless of the hard decision. Then, we explore novel estimation schemes, which are closely related to the hard decision.

#### 4.2.1. Analysis

With the quadratic estimation cost $C\left(x,\hat{x}\right)={\tilde{x}}^{\prime }\tilde{x}$, where $\tilde{x}=x-\hat{x}$, and based on the denotations in the previous part, the Bayesian tracking result is given by

(10) $$\begin{array}{cc} {\hat{x}}_{k|k} & =E(x_{k}|z^{k})\\ & =\sum_{i=1}^{N_{c}}E\left(x_{k}|c_{i},z^{k}\right)P\left\{c_{i}|z^{k}\right\} \end{array}$$

where $P\{c_{i}|z^{k}\}$ is the posterior class probability of $c_{i}$, which will be presented later. $E(x_{k}|c_{i},z^{k})$ is the state estimate under class $c_{i}$, defined by

(11) $$E\left(x_{k}|c_{i},z^{k}\right)=\sum_{j=1}^{r_{i}}E\left(x_{k}|m_{k}^{ij},c_{i},z^{k}\right)P\left\{m_{k}^{ij}|c_{i},z^{k}\right\}.$$

Here, $E(x_{k}|m_{k}^{ij},c_{i},z^{k})$ is state estimate based on model $m_{k}^{ij}$ under class $c_{i}$, and $P\{m_{k}^{ij}|c_{i},z^{k}\}$ is the corresponding probability of model $m_{k}^{ij}$ under class $c_{i}$. To improve the overall tracking performance of the $E(x_{k}|c_{i},z^{k})$, the well-known interacted multiple model (IMM) approach is adopted here. Details about the IMM estimator is presented in Table 1.

Under the linear Gaussian assumption, and for class $c_{i}$, both the model-based state estimate $E(x_{k}|m_{k}^{ij},c_{i},z^{k})$ and the likelihood $p(z_{k}|z^{k-1},c_{i})$ can be obtained using the Kalman filter. Based on the hybrid system consists of the evolution model (2) and the measurement model (3), the estimator at time *k* contains two main steps: prediction and update. Specifically, the predicted state and the corresponding mean square error (MSE) is:

$$\begin{array}{cc} {\hat{x}}_{k|k-1} & =F_{k-1}{\hat{x}}_{k-1|k-1}\\ P_{k|k-1} & =F_{k-1}P_{k-1|k-1}F_{k-1}^{T}+\Gamma_{k-1}Q_{k-1}\Gamma_{k-1}^{T} \end{array}$$

Then, with new measurement available, we can update the state and its corresponding MSE, as follows:

(12) $$\begin{array}{cc} {\hat{x}}_{k|k}= & {\hat{x}}_{k|k-1}+P_{k|k-1}H_{k}^{T}{\left(H_{k}P_{k|k-1}H_{k}^{T}+R_{k}\right)}^{-1}\\ & \times \left(z_{k}-H_{k}{\hat{x}}_{k|k-1}\right)\\ P_{k|k}= & P_{k|k-1}-P_{k|k-1}H_{k}^{T}{\left(S_{k}\right)}^{-1}H_{k}P_{k|k-1} \end{array}$$

Here, the updated state ${\hat{x}}_{k|k}$ work as $E(x_{k}|m_{k}^{ij},c_{i},z^{k})$ in our HSTC method.

In addition to the state estimate, another important issue is the measurement likelihood in (6). Under the linear Gaussian assumption, $p(z_{k}|z^{k-1},c_{j})$ can by calculated by:

(13) $$\begin{array}{c} p\left(z_{k}|z^{k-1},c_{j}\right)=e^{-{\tilde{z}}_{k}^{\prime }S_{k}^{-1}{\tilde{z}}_{k}/2}/\sqrt{2\pi \det\left(S_{k}\right)} \end{array}$$

in which the measurement residual ${\tilde{z}}_{k}$ is given by

$${\tilde{z}}_{k}=z_{k}-H_{k}{\hat{x}}_{k|k-1}$$

and the covariance $S_{k}$ is

$$S_{k}=H_{k}P_{k|k-1}H_{k}^{T}+R_{k}$$

**Remark 2:** *Generally, without considering the hard decision, the HSTC estimation is actually a layered multiple model solution with information fusion on two levels. The outer layer is the combination estimates results from different classes. The inner layer is the estimate under each target class. With this HSTC method, both tracker (10) and classifier (6) can be obtained simultaneously by accounting for the mutual information exchange between them [28].*

#### 4.2.2. Hard Decision Related HSTC Estimation

Denote by $t_{d}$ the hard decision time. As decision is closely related to estimation in HSTC, with respect to $t_{d}$, we split the whole time horizon into three parts: before $t_{d}$, at $t_{d}$ and after $t_{d}$.

##### Before $t_{d}$

Before the hard decision is made, both tracking and classification are concerned. The HSTC classifier and tracker and given in (6) and (10), respectively. For a clearer description, we present them again:

HSTC classifier:

$$P\left\{c_{i}|z^{k}\right\}=\frac{1}{\delta }p\left(z_{k}|z^{k-1},c_{i}\right)P\left\{c_{i}|z^{k-1}\right\}$$

HSTC tracker:

$${\hat{x}}_{k|k}=\sum_{i=1}^{N_{c}}E\left(x_{k}|c_{i},z^{k}\right)P\left\{c_{i}|z^{k}\right\}$$

Intuitively, estimation (for tracking) is a combination of the estimation results conditioned on all possible classes while decision (for classification) is a soft probability. In general, before hard decision is made, tracking and classification are obtained simultaneously by effectively using their mutual-effect. Specifically, classification affect tracking through the posterior class probability $P\{c_{i}|z^{k}\}$ while tracking affect classification through $p(z_{k}|z^{k-1},c_{i})$.

##### At $t_{d}$

At $t_{d}$, the hard decision is made, which is to “select” one class from all possible classes following SPRT. Accordingly, estimation is conditioned on decision, given by:

$${\hat{x}}_{k|k}^{i}=E\left(x_{k}|c_{i},z^{k}\right)$$

given by (11) with *i* being the selected class.

If the decision at $t_{d}$ is correct, we can get better tracking performance since only the true models are used for estimation and the computational burden will also significantly lowered. Although incorrect decision will deteriorate the estimation performance, on average, estimation would perform good since the decision performance is guaranteed by SPRT. This verifies that tracking and classification are closely linked.

##### After $t_{d}$

After the hard decision is made, we just need to do target tracking. Once the hard decision $D^{i}$ is made, it is unchangeable. Therefore, from $t_{d}$ on, the HSTC estimation is ${\hat{x}}_{k|k}^{i}$ and decision will always be $D^{i}$. In other words, terminating combination of the class-dependent estimates, and output the estimate corresponding to the hard decision $D^{i}$.

**Remark 3**: *Note that our HSTC method always have dual goals: tracking and classification. Before the hard decision is made, we take advantage of the two-way dependence between tracking and classification to achieve better performance of both. Once the hard decision is made, we output the decision and the corresponding estimation result simultaneously. After the hard decision, decision will not change and estimation is the one under decided class. It is worthwhile to mention that the HSTC algorithm significantly differs from the decision-based maneuvering target tracking (MTT) problem. MTT is a pure tracking problem, in which decision is secondary and assists estimation to achieve better tracking performance.*

**Remark 4**: *In this paper, we consider the simultaneous tracking and classification of the single target without measurement uncertainties. Thus, there is no data association problem, which is of paramount importance for multiple target tracking and classification. Actually, the proposed HSTC method can be also extended to the case for multiple target tracking and classification by adopting some data association techniques, i.e., joint probabilistic data association (JPDA). Once we determine the state estimate under each target class and the corresponding likelihood of the target class, the proposed HSTC still works.*

## 5. Illustrative Examples

In this Section, two simple but representative joint tracking and classification problem with hard decision are presented for illustration. Suppose there is only one target with two possible types $c_{1}$ and $c_{2}$, i.e., a fighter and an airliner. Our goal is to simultaneously track and identify the target, and take corresponding actions after the hard decision is made. Note that this paper considers the military application of the JTC problem. It is difficult to obtain real data in a battlefield environment. Therefore, we use the simulated data in the following.

It is worthwhile to mention that in this paper, target tracking is based on radar data, which differs from visual tracking. In visual tracking, the number of measurements (e.g., pixels in a frame) in much larger and the noise is much lower than in our case, so a lot of specific object information can be extracted (features including color, edges) extracted from the images [23,32,33]. However, this information cannot be obtained for tracking using radar data, where only dozens of position measurements may be available.

In example 1, classes differ from each other in maneuverability. The single model HSTC method is adopted using only kinematic measurements. In example 2, classes differ from each other in both maneuverability and attribute. The multiple model HSTC method is adopted using both kinematic and attribute measurements.

### 5.1. Example 1

With the linear motion assumption, the target state evolves according to the following evolution model:

(14) $$\left[\begin{array}{c} x_{k+1}\\ {\dot{x}}_{k+1} \end{array}\right]=\left[\begin{array}{cc} 1 & T\\ 0 & 1 \end{array}\right]\left[\begin{array}{c} x_{k}\\ {\dot{x}}_{k} \end{array}\right]+\left[\begin{array}{c} \frac{1}{2}T^{2}\\ T \end{array}\right]u_{k}^{i}+\left[\begin{array}{c} \frac{1}{2}T^{2}\\ T \end{array}\right]w_{k}$$

The only difference between the two classes is the control input $u_{k}^{i}$. Specifically, class 1 has $u_{k}^{i}=1g$ while class 2 has $u_{k}^{i}=1.5g$. Here, *g* denotes one gravitational acceleration.

The kinematic measurement model is given by

(15) $$z_{k}=x_{k}+v_{k}$$

The standard deviations of the process noise $w_{k}$ and measurement noise $v_{k}$ were set as q=10 m/s${}^{2}$ and r=100 m/s, respectively. Sampling time T=1 s. The simulation results were based on the average of 5000 Monte Carlo runs. The ground truth was generated from a Gaussian distribution $N({\bar{x}}_{0},P_{0})$ with ${\bar{x}}_{0}=[8000\;$ m,200 m/s${]}^{\prime }$ and $P_{0}=diag\left[10^{6}\;m^{2},10^{2}\;m^{2}/s^{2}\right]$. The simulation results were obtained by 5000 Monte Carlo runs, and the true target class was 1.

Table 2 shows the decision performance of the proposed HSTC method. It can be seen that the actual error rate is smaller than the controlled value. For the controlled type I and type II error α=β=0.01, the actual error rate is only 0.0086; for α=β=0.03, it is only 0.0256. The third column shows the time instant when the hard decision is made in HSTC, which is critical in joint tracking and classification with hard decision. It took HSTC only 45.2548 s on average to identify the class label for α=β=0.03 and only 34.7372 s on average for α=β=0.01. In general, Table 2 verifies that on the premise of ensuring the decision error rate less than the controlled value, the hard decision can be made timely.

Table 3 shows that HSTC saves about thirty-five percent of the calculation compared with STC, which is consistent with the theoretical analysis in the previous part. In HSTC, when the hard decision is made on class 1, all the calculation related to class 2 will be terminated, and thus the computational burden will surely be reduced. This makes HSTC more applicable in practice.

**Figure 1:**
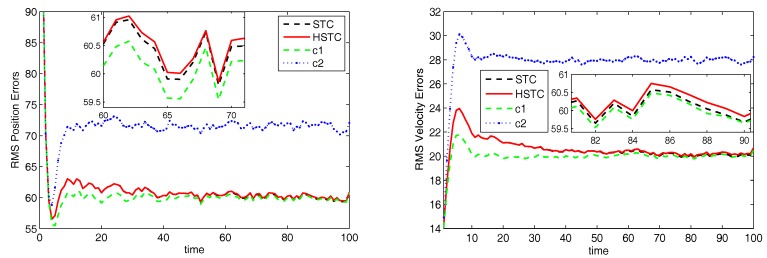
RMS error of position and velocity.

Figure 1 shows the estimation performance of HSTC. The tracking errors of STC and HSTC are both between those of using classes c1 and c2, and as time goes on, they approach the performance under true class quickly. Note that STC is slightly better than HSTC finally, resulting from decision errors. Actually, STC only pursues the tracking performance. As the weighted sum of estimates under both classes, STC has the optimal estimation performance in the sense of minimum mean square error. However, HSTC has dual goal: classification (make a correct and quick decision) and tracking (have small estimation error), and we need to achieve good classification and tracking performances simultaneously.

It is worthwhile mentioning that Example 1 is adopted as a simple scenario for illustration. In this example, each target has only one type of maneuvering mode, which is the only characteristic that distinguishes the targets. We do not adopt the multiple model approach because in this simple case, the errors caused by inappropriate modeling will be eliminated, and thus it can only result from the HSTC method. Actually, when multiple models are taken into account, similar conclusions can be obtained, which will be shown next.

**Remark 5**: *This example shows that the proposed HSTC method satisfies the requirements of practical JTC problems involving hard decision. What is more important is that the superiority of HSTC is also demonstrated in three aspects: a) the decision error rate is within the controlled value; b) the hard decision can be obtained timely; c) the HSTC estimation also performs good, which is close to the optimal MSE estimation.*

### 5.2. Example 2

In this example, we consider a JTC problem with hard decision using both kinematic and attribute measurements. There is no unified model description of a target feature. For example, radar cross section (RCS) is a function of the orientation of the target and its range, size, and type. Infrared (IR) can provide the shape features such as target spatial distribution and target area. Electronic support measurement (ESM) exploits target EM emission to supply target identity information. In this paper, we adopt the ESM measurement similar to [24]. Each target has several types of emitters, which can be used as an attribute feature. At every time instant, an emitter is either “on” or “off”, and this state switches according to a Markov chain. More details can be found in [24].

Denote by $z_{k}^{x}$ the kinematic measurement and $z_{k}^{c}$ the attribute measurement, respectively. $f_{k}$ is the target attribute feature, which contains all the attribute information and can evolve over time. Then, the joint measurements can be modeled by the conditional pdf-pmf

(16) $$p(z_{k}^{x},z_{k}^{c}|x_{k},f_{k})$$

For simplicity, assume that the two measurement processes are conditionally independent:

(17) $$p\left(z_{k}^{x},z_{k}^{c}|x_{k},f_{k}\right)=p\left(z_{k}^{x}|x_{k}\right)p\left(z_{k}^{c}|f_{k}\right)$$

This is usually reasonable since $z_{k}^{x}$ only depends on the target state $x_{k}$ and $z_{k}^{c}$ only depends on the target feature $f_{k}$.

Each class is modeled by multiple dynamic models. Target motion model is of the same form as before except that $u_{k}$ for class *i* belongs to model set $M^{i}$, i=1,2, where $M^{1}=\left\{0,+g,-g\right\},\;M^{2}=\left\{0,+g,-g,+5g,-5g\right\}$. The initial model probabilities for each class are $\mu_{0}^{1}=\left\{1/3,1/3,1/3\right\}$ and $\mu_{0}^{2}=\left\{1/5,1/5,1/5,1/5,1/5\right\}$. For each run the true target was still class 1, and the truth state was still generated from a Gaussian distribution as in Example 1. For the attribute evolution and measurement model, suppose class 1 has emitter $E^{1}$ and class 2 has emitter $E^{2}$. To describe the independently emitter usage process, the transition probability matrices for these two classes are the same as $\Phi^{1}$ and $\Phi^{3}$ in [28]. The measurement process is p(declare $E^{j}|$declare $E^{i})$ equals to 0.8 for i=j and 0.2 otherwise. For more details about the evolution and measurement of the attribute data, please refer to [24].

The IMM estimation is adopted in this example. In each class, the models switch according to the transition probability matrix (TPM). The TPM for class 1 target is given by

$$\Pi^{1}=\left[\begin{array}{ccc} 0.9 & 0.05 & 0.05\\ 0.15 & 0.85 & 0\\ 0.15 & 0 & 0.85 \end{array}\right]$$

for class 2 target is given by

$$\Pi^{2}=\left[\begin{array}{ccccc} 0.9 & 0.025 & 0.025 & 0.025 & 0.025\\ 0.15 & 0.85 & 0 & 0 & 0\\ 0.15 & 0 & 0.85 & 0 & 0\\ 0.15 & 0 & 0 & 0.85 & 0\\ 0.15 & 0 & 0 & 0 & 0.85 \end{array}\right]$$

The results were based on 5000 Monte Carlo runs. Sampling time T=1 s, the covariance of process noise and measurement noise were $Q=10^{2}$ (m/s${}^{2}$)${}^{2}$ and $R=10^{4}\;$(m/s)${}^{2}$, respectively. Controlled type I and type II errors α=β=0.03.

Table 4 shows the decision performance of the proposed HSTC method using both kinematic and attribute measurements. When compared with Table 2, Table 4 shows that given the same type I and type II errors, HSTC using both kinematic and attribute measurements can make a decision much quicker with smaller actual error rate. Take α=β=0.3 as an example, it takes HSTC with multisensor data only 6.2720 s to identify the class label with only 0.0010 actual error rate, while 45.2548 s with 0.0256 actual error rate for HSTC with only radar measurement. This demonstrates that the performance of classification is significantly improved with the help of the attribute measurements.

Table 5 shows that HSTC can save nearly half of the computational load compared to STC. However, when comparing Table 5 with Table 3, i.e., the computational load with kinematic data only, we can find that both the computation complexity of STC and HSTC increase due to the introduction of the attribute measurements.

**Figure 2:**
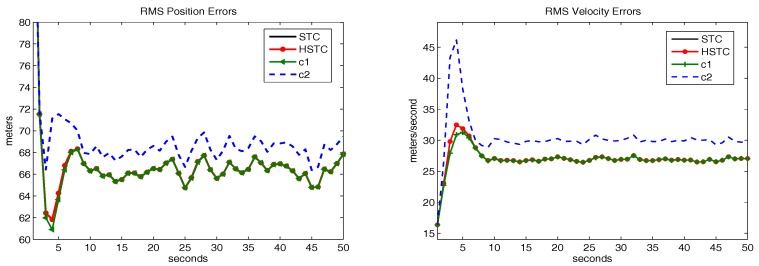
RMS error of position and velocity in Example 2.

Figure 2 shows the tracking performance of HSTC using both kinematic and attribute measurements. Compared with Figure 1, similar rule can be found in Figure 2. However, with the help of attribute measurements, all tracking error curves converge to their steady state values more quickly. Besides, the gap between the steady state value and the bound is much smaller in Figure 2 than in Figure 1.

**Remark 6**: *Generally speaking, the HSTC method can meet the demand of the joint tracking and classification problem with hard decision. It can explicitly output the hard decision of the target class. Meanwhile, it accounts for both the timeliness of hard decision, the decision error, and also the corresponding estimation performance.*

## 6. Conclusions

To solve the practical JTC problem with hard decision, this paper proposes a Hard decision-based STC (HSTC) method, in which SPRT is adopted for decision making. The proposed HSTC method takes advantage of both the SPRT and the STC method. Specifically, on the premise that decision error rate is within the acceptable range, the HSTC method can make an explicit hard decision about the class label as soon as possible. For estimation, different estimation strategies are provided by fully accounting for the mutual information exchange between tracking and classification.

In general, with the proposed HSTC method, all the decision timeliness, decision error, and the estimation performances are considered. Simulation verifies that HSTC cannot only meet the demands of JTC problem with hard decision but also performs good in both tracking and classification. In this paper, we only consider the case of single target with binary target classes. For multiple targets simultaneously detection, tracking and classification with multi-classes is under further research.

## Figures and Tables

**Table 1:** One cycle of IMM estimator.

1. Model-conditioned re-initialization (for i=1,2,⋯,M):	
Predicted mode probability:	$\mu_{k|k-1}^{\left(i\right)}≜P\left\{m_{k}^{\left(i\right)}|Z^{k-1}\right\}=\sum_{j}\pi_{ji}\mu_{k-1}^{\left(j\right)}$
Mixing weight:	$\mu_{k-1}^{j|i}≜P\left\{m_{k-1}^{\left(j\right)}|m_{k}^{\left(i\right)},Z^{k-1}\right\}=\pi_{ji}\mu_{k-1}^{\left(j\right)}/\mu_{k|k-1}^{\left(i\right)}$
Mixing estimate:	${\bar{x}}_{k-1|k-1}^{\left(i\right)}≜E\left[x_{k-1}|m_{k}^{\left(i\right)},Z^{k-1}\right]=\sum_{j}{\hat{x}}_{k-1|k-1}^{\left(j\right)}\mu_{k-1}^{j|i}$
Mixing covariance:	${\bar{P}}_{k-1|k-1}^{\left(i\right)}=\sum_{j}\left[P_{k-1|k-1}^{\left(j\right)}+\left({\bar{x}}_{k-1|k-1}^{\left(i\right)}-{\hat{x}}_{k-1|k-1}^{\left(j\right)}\right)\left({\bar{x}}_{k-1|k-1}^{\left(i\right)}-{\hat{x}}_{k-1|k-1}^{\left(j\right)}\right)\right]\mu_{k-1}^{j|i}$
2. Model-conditioned filtering (for i=1,2,⋯,M):	
Predicted state:	${\hat{x}}_{k|k-1}^{\left(i\right)}=F_{k-1}^{\left(i\right)}{\bar{x}}_{k-1|k-1}^{\left(i\right)}+G_{k-1}^{\left(i\right)}{\bar{w}}_{k-1}^{\left(i\right)}$
Predicted covariance:	$P_{k|k-1}^{\left(i\right)}=F_{k-1}^{\left(i\right)}{\bar{P}}_{k-1|k-1}^{\left(i\right)}{\left(F_{k-1}^{\left(i\right)}\right)}^{\prime }+G_{k-1}^{\left(i\right)}Q_{k-1|k-1}^{\left(i\right)}{\left(G_{k-1}^{\left(i\right)}\right)}^{\prime }$
Measurement residual	${\tilde{z}}_{k}^{\left(i\right)}=z_{k}-H_{k}^{\left(i\right)}{\hat{x}}_{k|k-1}^{\left(i\right)}-{\bar{v}}_{k}^{\left(i\right)}$
Residual covariance	$S_{k}^{\left(i\right)}=H_{k}^{\left(i\right)}P_{k|k-1}^{\left(i\right)}{\left(H_{k}^{\left(i\right)}\right)}^{\prime }+R_{k}^{\left(i\right)}$
Filter gain	$K_{k}^{\left(i\right)}=P_{k|k-1}^{\left(i\right)}{\left(H_{k}^{\left(i\right)}\right)}^{\prime }{\left(S_{k}^{\left(i\right)}\right)}^{-1}$
Updated state	${\hat{x}}_{k|k}^{\left(i\right)}={\hat{x}}_{k|k-1}^{\left(i\right)}+K_{k}^{\left(i\right)}{\tilde{z}}_{k}^{\left(i\right)}$
Updated covariance	$P_{k|k}=\sum_{i}\left[P_{k|k}^{\left(i\right)}+\left({\hat{x}}_{k|k}-{\hat{x}}_{k|k}^{\left(i\right)}\right){\left({\hat{x}}_{k|k}-{\hat{x}}_{k|k}^{\left(i\right)}\right)}^{\prime }\right]\mu_{k}^{\left(i\right)}$
3. Model probability update (for i=1,2,⋯,M):	
Model likelihood:	$L_{k}^{\left(i\right)}≜p\left[{\tilde{z}}_{k}^{\left(i\right)}|m_{k}^{\left(i\right)},Z^{k-1}\right]=N\left({\tilde{z}}_{k}^{\left(i\right)};0,S_{k}^{\left(i\right)}\right)$
Mode probability:	$\mu_{k}^{\left(i\right)}≜\frac{\mu_{k}^{\left(i\right)}L_{k}^{\left(i\right)}}{\sum_{j}\mu_{k}^{\left(j\right)}L_{k}^{\left(j\right)}}$
4. Estimation fusion	
Overall estimate:	${\hat{x}}_{k|k}=\sum_{i}{\hat{x}}_{k|k}^{\left(i\right)}\mu_{k}^{\left(i\right)}$
Overall covariance:	$\mu_{k-1}^{j|i}≜P\left\{m_{k-1}^{\left(j\right)}|m_{k}^{\left(i\right)},Z^{k-1}\right\}=\pi_{ji}\mu_{k-1}^{\left(j\right)}/\mu_{k|k-1}^{\left(i\right)}$

**Table 2:** Simulation results of HSTC.

α,β	Actual Error Rate	Average Decision Time
0.01	0.0086	45.2548 s
0.03	0.0256	34.7372 s

**Table 3:** Average computational load per run (α=β=0.03).

STC	HSTC
$1.3289\times 10^{-2}$ s	$0.8677\times 10^{-2}$ s

**Table 4:** Simulation results of HSTC (kinematic + attribute).

α,β	Actual Error Rate	Average Decision Time
0.01	0.0002	7.6310 s
0.03	0.0010	6.2720 s

**Table 5:** Average computational load per run (kinematic + attribute) (α=β=0.03).

STC	HSTC
$2.3034\times 10^{-2}$ s	$1.238\times 10^{-2}$ s
